# Nitrogen form and mycorrhizal inoculation amount and timing affect flavonol biosynthesis in onion (*Allium cepa* L.)

**DOI:** 10.1007/s00572-017-0799-3

**Published:** 2017-09-26

**Authors:** Mohanna Mollavali, Henrike Perner, Sascha Rohn, Peer Riehle, Franziska S. Hanschen, Dietmar Schwarz

**Affiliations:** 1 Vegetable Physiology Laboratory, Department of Horticulture, University of Tabriz, Tabriz, Iran; 2 Leibniz Institute for Vegetable and Ornamental Crops, Theodor-Echtermeyer-Weg 1, 14979 Großbeeren, Germany; 3 Institute of Food Chemistry, Hamburg School of Food Science, University Hamburg, Grindelallee 117, 20146 Hamburg, Germany

**Keywords:** Ammonium, Chalcone synthase, Flavonol synthase, Nitrate, Quercetin, Phenyl alanine lyase

## Abstract

Mycorrhizal symbiosis is known to be the most prevalent form of fungal symbiosis with plants. Although some studies focus on the importance of mycorrhizal symbiosis for enhanced flavonoids in the host plants, a comprehensive understanding of the relationship still is lacking. Therefore, we studied the effects of mycorrhizal inoculation of onions (*Allium cepa* L.) regarding flavonol concentration and the genes involved in flavonol biosynthesis when different forms of nitrogen were supplied. We hypothesized that mycorrhizal inoculation can act as a biotic stress and might lead to an increase in flavonols and expression of related genes. The three main quercetin compounds [quercetin-3,4′-di-*O*-*β*-d-glucoside (QDG), quercetin-4′-*O*-*β*-d-glucoside (QMG), and isorhamnetin-4′-*O*-*β*-d-glucoside (IMG)] of onion bulbs were identified and analyzed after inoculating with increasing amounts of mycorrhizal inocula at two time points and supplying either predominantly NO_3_^−^ or NH_4_^+^ nitrogen. We also quantified plant dry mass, nutrient element uptake, chalcone synthase (*CHS*), flavonol synthase (*FLS*), and phenyl alanine lyase (*PAL*) gene expression as key enzymes for flavonol biosynthesis. Inoculation with arbuscular mycorrhizal fungi (highest amount) and colonization at late development stages (bulb growth) increased QDG and QMG concentrations if plants were additionally supplied with predominantly NH_4_^+^. No differences were observed in the IMG content. RNA accumulation of *CHS*, *FLS*, and *PAL* was affected by the stage of the mycorrhizal symbiosis and the nitrogen form. Accumulation of flavonols was not correlated, however, with either the percentage of myorrhization or the abundance of transcripts of flavonoid biosynthesis genes. We found that in plants at late developmental stages, RNA accumulation as a reflection of a current physiological situation does not necessarily correspond with the content of metabolites that accumulate over a long period. Our findings suggest that nitrogen form can be an important factor determining mycorrhizal development and that both nitrogen form and mycorrhizas interact to influence flavonol biosynthesis.

## Introduction

Onion (*Allium cepa* L.) is one of the world’s oldest and most widely cultivated vegetables which has great economic importance, particularly in Asia and Europe (Griffiths et al. 2002). Previous studies on the health benefits of onion reported that bulbs are rich in two main groups of chemical compounds: *S*-alk(en)yl-l-cysteine sulfoxides and flavonoids (Crozier et al. 1997; Price et al. 1997; Griffiths et al. 2002). Flavonoids are a diverse class of polyphenolic compounds. In onion, the flavonols quercetin, isorhamnetin, and kaempferol derivatives are present; it is a subgroup of flavonoids (Bilyk et al. 1984). They typically occur glycosylated with sugars such as glucose. The main flavonols in onion have been determined as quercetin-3,4′-di-*O*-*β*-d-glucoside (QDG) and quercetin-4′-*O*-*β*-d-glucoside (QMG), both making up to 80–85% of the total flavonoid content (Price and Rhodes 1997; Rhodes and Price 1996). Flavonoids have received considerable attention based on their many functions in plants, including protection against ultraviolet radiation, regulation of auxin transport, and modulation of flower color (Buer et al. 2010). They also may act as signaling molecules involved in plant defense mechanisms against fungal pathogen attack or directly as plant defense agents (Bi et al. 2007; Mandal et al. 2010). Chalcone synthase (CHS) is a key enzyme in the flavonoid biosynthesis pathway and therefore is involved with the biosynthesis of dihydroquercetin where the pathway divides into the formation of the anthocyanidin cyanidin and the flavonol quercetin. Quercetin is formed by flavonol synthase (FLS) and further transformed to the glucosides by the appropriate quercetin-4′-*O*-glucosyl transferase and quercetin-3,4′-*O*-glucosyl transferase (Materska 2008; Petrussa et al. 2013).

Onions are considered as highly mycorrhiza-dependent plants because they have a low root/shoot ratio and low phosphorus inflow rates (Augé and Moore 2005). Arbuscular mycorrhizal fungi (AMF) are known to significantly influence the composition of flavonoids in onions and other plants (Ling-Lee et al. 1977; Ponce et al. 2004; Perner et al. 2008). AMF contact with host plant roots initiates defense mechanisms (Harrison and Dixon 1994; Bi et al. 2007). In consequence, production of flavonoid compounds and antioxidant enzyme activities increase (Ling-Lee et al. 1977; Devi and Reddy 2002). The *CHS* gene, a key enzyme in flavonoid biosynthesis, has been induced by AMF in *Medicago truncatula* (Bonanomi et al. 2001). The compounds derived from induction of *CHS* expression are responsible for numerous functions, e.g., antimicrobial effect, insecticidal effect, or antioxidant activity by transforming phytoalexins (Dao et al. 2011). However, it is not clear if the enhancement of flavonoid biosynthesis by AMF is a temporary or a persistent effect.

Nitrogen supply affects bulb yield, bulb grade, firmness, maturity, and storability of onions (Charron et al. 2001; Mozumder et al. 2007). Moreover, nitrogen deficiency and nitrogen form can affect the accumulation of secondary metabolites as expressed by the carbon/nutrient balance hypothesis (Herms and Matson 1992; Scheible et al. 2004; Nguyen and Niemeyer 2008). It postulates that the status of carbon and nitrogen of plants controls the concentrations of secondary metabolites (Hamilton et al. 2001). It has been shown that nutrient stress, such as nitrogen deficiency, leads to an increase in flavonoid production in apple (*Malus domestica*) (Awad and de Jager, 2002, Strissel et al. 2005), *Labisia pumila* (Ibrahim et al. 2012), and leaves of mature tomatoes (*Solanum lycopersicum*) (Stewart et al. 2001). Furthermore, nutrient stress resulted in the expression of flavonoid pathway regulators in *Arabidopsis thaliana* (Lea et al. 2007). In connection with AMF colonization, predominant NH_4_^+^ nutrition increased the QMG concentration in onion bulbs while predominant NO_3_^−^ nutrition increased the QDG concentration when *Allium* roots were highly colonized (Perner et al. 2008).

Measurement of mRNAs of the key enzymes of flavonoid biosynthesis, phenylalanine ammonium lyase (PAL), and CHS, in the root cells of *Phaseolus vulgaris* showed increased accumulation of the enzymes in roots colonized with AMF (Kristopher and Anderson 1996). Taken together, it can be concluded that nitrogen deficiency and AMF root colonization cause an increase of flavonoid production.

For the present study, we hypothesized that AMF colonization can act as a biotic stress and consequently enhance flavonols as a defense mechanism, as well as inducing expression of responsible genes involved in flavonol biosynthesis. To test this hypothesis, we analyzed three main flavonols and the expressions of three key genes (*PAL*, *CHS*, and *FLS*) triggering flavonol biosynthesis in onions. Different time points after AMF inoculation were examined in order to avoid that the induction time was missed or that plant and fungi are habituated to the symbiosis. Nitrogen supply affects AMF colonization of the host plant through direct effects involving morphological changes in AMF and indirect effects by reduced C allocation from plant to fungus (Blanke et al. 2005, Olsson et al. 2005). Moreover, different nitrogen forms have an impact on the flavonol concentration (Fallovo et al. 2011). So, we also tested whether even low concentrations of AMF inoculum induce and increase flavonol concentration in order to determine if such a mycorrhizal effect would be persistent. Therefore, in the present experiments, the effects on the flavonol profile and on key enzymes in the flavonol biosynthesis pathway in onion of different inoculum concentrations, different times of inoculation, and different nitrogen forms were investigated. Because the onion variety “Stuttgarter Riesen” has high total flavonol content (unpublished data) versus other cultivars (Beesk et al. 2010) and frequently is used in European horticultural practice, we selected this cultivar for our experiment.

## Material and methods

### Onion cultivation and treatments

A pot experiment with eight onion sets per pot was carried out from 22 April till 18 September 2013 in greenhouse facilities at Großbeeren, Germany (long. 13° 20′ E; lat. 51° 22′ N). The experiment was arranged in a completely randomized design with three fully crossed factors and six replications. The first factor consisted of arbuscular mycorrhizal fungi (volume fraction of 0, 0.3, and 3%), the second factor of two inoculation dates (0 and 65 days after sowing), and the third factor of two nitrogen supply forms (95% NO_3_^−^/5% NH_4_^+^ and 25% NO_3_^−^/75% NH_4_^+^). Onion seeds (*A. cepa*, cv. Stuttgarter Riesen) were suspended in 10% aqueous H_2_O_2_ for 10 min to sterilize the surface. Subsequently, they were washed three times with distilled water, moistened with CaSO_4_ solution, and sown directly into pots (*Ø* = 19 cm, 2.3 L) filled with sterilized quartz sand (particle size 0.5–1 mm; Ottendorf-Okrilla GmbH, Lausnitz, Germany). Before using, the quartz sand was sterilized by heating for 2 days at 95 °C and afterwards was cooled. A commercial AMF inoculum (INOQ GmbH, Schnega, Germany) comprising *Funneliformis mosseae* and *Rhizophagus irregularis* (as identified by the company) was equally mixed with the quartz sand at the previously described volume fractions. Sterilized inoculum (autoclaved at 121 °C for 20 min) was applied likewise at volume fractions of 3 and 2.7% to the 0 and 0.3% treatments, respectively. In this way, all treatments had comparable inoculum amounts totalling 3%. In addition, the liquid of non-sterilized inoculum was filtered before sterilization (589/3 blue ribbon paper filter, Schleicher and Schuell Bioscience GmbH, Dassel, Germany) and added to the appropriate pots. This procedure was carried out to supply similar amounts of nutrients and microorganisms except AMF to all treatments. Half of the pots of the experiment were inoculated before seeding and the other half 65 days later, when the plants started bulbing. At the second inoculation date, all plants were thoroughly rooted and were transferred with their whole substrate into large pots (*Ø* = 26 cm, 6 L). In total, 48 pots were inoculated with live inoculum as described above, while 24 got the same amount of sterilized inoculum.

All 72 pots were kept in a heated greenhouse for 44 days at a mean temperature of 18 °C. Seedlings were watered with a fifth-strength modified Hoagland solution at pH 5.6 (Hoagland and Arnon 1938). When the plants reached the third-leaf stage, the pots were transferred to an open-sided greenhouse. After 7 days of adaption, the fertilizer treatment started (51 days after seeding): plants were watered with a third-strength Hoagland solution. Nitrogen was supplied at two NO_3_^−^/NH_4_^+^ ratios: 95/5% (predominant NO_3_^−^ supply) or 25/75% (predominant NH_4_^+^ supply). The nutrient solution contained the following: (mmol) NO_3_^−^7.03/1.85, NH_4_^+^ 0.37/5.5, K^+^ 2.7, PO_4_^3−^0.4, Mg^2+^ 1.6, SO_4_^2−^1, Ca^2+^ 3.6, and (μmol) Fe^2+^ 55 Mn^3+^ 2.5, Zn^2+^ 0.4, BO_3_^3−^18, Cu^2+^ 0.25, MoO_4_^2+^ 0.18, Cl^−^8.84/14.41. Nutrient solution was supplied until one-third of the added solution drained from the pots. A pH value of 5.6 was maintained by adding NaOH and MES buffer in a concentration of 2 mmol. Watering was reduced 14 days prior to harvest to let plants dry. Average air temperature in the open-sided greenhouse during this time was 21/17 °C (day/night) and reached values of maximum 39 °C and minimum 13 °C. The relative humidity was on average 54/68% (day/night). The mean daily photosynthetic active radiation was 29.2 mol m^−2^ with a daily minimum of 6 and a maximum of 48 mol m^−2^.

### Harvest

Plants were harvested 149 days after sowing, when 80% reached the soft neck stage. Six bulbs per pot were weighed including their dry skin to obtain total fresh mass and then were separated into two portions. Three bulbs were dried in an oven at 60 °C and dry mass was determined after 2 days. Bulb dry mass samples were ground in a centrifugal grinder with a 0.5-mm sieve and analyzed for total nitrogen, phosphorus, and potassium concentration. The outer skin of the remaining three bulbs was removed, and then, the bulbs were cut into small pieces. Thereafter, the samples were frozen at − 20 °C and later freeze-dried for flavonol analyses.

### Analyzing AM colonization and mineral element uptake

Roots randomly gathered from the pots at the final harvest date, 149 days after sowing, were washed and cut into 10 mm length, then cleared with 10% KOH solution incubated at 60 °C for 30 min. Samples were acidified with 2 N HCl for 2 min at room temperature and stained with 5% ink-acid solution for 40 min at 60 °C in an oven. Thereafter, roots were rinsed a few times with tap water and were kept in 90% lactic acid overnight for destaining (Koske and Gemma 1989). Fifty root segments were put on slides and the percentage of root length colonization was determined with a microscope (Zeiss, Stemi2000, Göttingen, Germany) at ×50 using the gridline intersection method (Giovannetti and Mosse 1980), modified from Perner et al. (2007). Two hundred gridline intersections were assessed per sample and the presence of arbuscules, hyphae, and vesicles was used to determine AMF colonization.

Ground bulb material was dry-ashed and dissolved in 18.5% HCl. Phosphorus, potassium, and nitrogen concentrations were analyzed and measured following standardized procedures we briefly describe as follows. Potassium was analyzed with an atomic absorption spectrophotometer (Perkin Elmer 3300, Überlingen, Germany), and phosphorus was analyzed photometrically with an EPOS-Analyzer 5060 (Eppendorf, Hamburg, Germany). Nitrogen was determined after dry oxidation by the Dumas method (ElementarVario EL, Hanau, Germany). Contents of mineral elements analyzed were calculated by multiplying mineral element concentration by the bulb dry mass.

### Flavonol analyses using HPLC–DAD

The flavonoid profiles of the three major flavonols QDG, QMG, and isorhamnetin-4′-*O*-*β*-d-glucoside (IMG) were analyzed. High-performance liquid chromatography with diode-array detection (HPLC-DAD) analysis of the flavonols was performed as described by Beesk et al. (2010), with slight modifications. For the analysis of onion bulbs, 2.5 g of lyophilized, powdered onion samples was extracted with 50 mL aqueous methanol (volume faction of 70%) for 30 min under continuous stirring. After a filtration step (Whatman filter, *Ø*150 mm, 597½), 4 mL of the filtrate was dried under a gentle stream of nitrogen, subsequently diluted with 2 mL of water, and loaded onto a solid phase extraction column (Chromabond PA, 6 ml, 500 mg, Macherey–Nagel, Germany). The column was washed with 10 mL of water to remove sugars and other water-soluble compounds. The flavonols were eluted with 10 mL of a methanol/water/acetic acid mixture (volume fraction of 90:5:5). This dilution was used for the HPLC-DAD analysis (Smartline series system from Knauer GmbH, Berlin, Germany). The LPG (low pressure gradient) chromatography consisted of a Smartline manager (5050 series), pump (1000 series), autosampler (3950 series), and diode array detector (2600 series). The system was controlled by ClarityChrom 3.0 software (Knauer GmbH, Berlin, Germany). A binary gradient system based on Riehle et al. (2013) with eluent (A) 0.1% formic acid in water, eluent (B) 0.1% formic acid in acetonitrile was conducted on a Luna® 5 μm C18 100 Å (150 × 3.00 mm) column equipped with a C18 security guard (4 × 3.00 mm), both from Phenomenex Inc. (Aschaffenburg, Germany). Gradient elution was used for methanolic SPE eluates: 5% B isocratic (0–2 min), 5–10% B (2–6 min), 10–30% B (6–45 min), 30–95% B (45–55 min), 95% B isocratic (55–60 min), 95–5% B (60–65 min), and 5% isocratic (65–75 min). The flow rate was 0.6 mL/min and the column temperature was 21 °C. The detection was performed simultaneously at 280, 325, and 365 nm.

### *CHS*, *FLS*, and *PAL* gene expression analyses

Samples were taken 7 days after the second inoculation (65 days after seeding) because gene expression of chalcone, flavonol synthase, and PAL is detectable at the beginning of the formation of the symbiosis (Bonanomi et al. 2001). One bulb from each pot was harvested, immediately frozen in liquid nitrogen, and kept at − 80 °C until further analysis. Total RNA was extracted from bulb tissue derived from three replications (100 mg) with an RNeasy Plant MiniKit (QIAGEN, Hilden). First strand complementary DNA (cDNA) was synthesized from total RNA (2 μg) with M-MLV reverse transcriptase (Promega, Mannheim) and oligo d(T) primer (10 mmol) according to the manufacturer’s protocol. Gene-specific primers were designed using the Lasergene software (DNASTAR, Madison, WI, USA) as follows:

- *CHS* (forward): 5′-AGTGCGTGCGTGTTGTTTAT-3′,
- *CHS* (reverse): 5′-GAAGCACAACGGTCTCAACA -3′,
- *FLS* (forward): 5′-TTTGGAAGGGAAGAAGGCCT-3′,
- *FLS* (reverse): 5′-TGTGTACTCCTCGTTTGCCT-3′,
- *PAL* (forward): 5′-TTCTTGTACAGCATGCACTGA-3′,
- *PAL* (reverse): 5′-TATTCTATTAGGAACCACTGCA-3′,
- *ALL ITS1* (forward): 5َ´-TATGTTCCACTGGCAGGCTACTAT-3′,
- *ALL ITS1* (reverse): 5′-TGGAAATGGTTAACGCAGGAC-3′.

The resulting cDNA served as a template for PCR amplification. All PCRs were performed as follows: an initial denaturation of 1 min at 95 °C and 35 cycles of 95 °C for 30 s, 61 °C (*CHS1*) or 56 °C (*FLS1*) for 30 s, 72 °C for 10 s, and then a final 10-min extension at 72 °C. Seven microliters of PCR products was analyzed by 3% agarose gel electrophoresis. Real-time PCR (Applied Biosystems, 7500 Real-Time PCR System) was performed in a 10 μL reaction volume with 200 nmol/L for each primer and 2 × SensiMix SYBR Low-ROX (Bioline, Luckenwalde, Germany). Values represent gene transcript abundance of the three genes normalized to the reference gene *ALL ITS1* derived from three independent repetitions. RT-PCR reactions were performed in a 10 μL reaction mixture containing 1 μg template, 5 μL SYBER Green, 2 μL forward primer (200 nmol/L), 2 μL reverse primer (200 nmol/L). All PCRs were performed as previously described.

### Statistical analysis

Data were analyzed in accordance with the experimental design. The percentage root length colonized was determined for three replications and non-parametrically analyzed using Kruskal-Wallis ANOVA. Means were compared by Mann-Whitney *U* test at a significance level of *α* = 0.05. A three-way ANOVA was used to evaluate the treatment effects for all other characteristics at a significance level of *α* = 0.05. Means were compared with Duncan’s multiple range test. All statistical analyses were conducted with the “SPSS” computer software package (v. 18.0, SPSS, IBM, USA).

## Results

### Mycorrhizal colonization

All inoculated plants were colonized with AMF except those that received a low amount (0.3 *v*/*v* %) of inoculum at predominant NH_4_^+^ supply (Table 1). The percentage root length colonized ranged from 0 to 74.7% among the treatments. The highest percentage root length colonized was found with first inoculation (0 day), highest amount of inoculum (3%), and predominant NO_3_^−^. The three factors, nitrogen form and amount and time of inoculation, however, seem to interact in their effects on mycorrhizal colonization. Plants fertilized predominantly with NO_3_^−^ showed a significantly higher percentage root length colonized than those fertilized predominantly with NH_4_^+^ and with increasing amounts of AMF inoculum. Percentage root length colonized was 0 or low in those pots treated with predominant NH_4_^+^ independent of the amount and date of live inoculum supplied. Roots of all non-inoculated plants remained free of AMF colonization.

**Table 1:** Effect of mycorrhizal inoculation (AMF), date of inoculation (days after seeding), and nitrogen (N) form on the percentage root-length colonized (PRC) in onion plants (*cv.* Stuttgarter Riesen) analyzed after a growing period of 149 days in an orthogonal experimental scheme with six replications. Means ± standard deviation (SD) followed by the same letter do not differ significantly according to Mann-Whitney *U* test. Probability levels written in italics indicate significant differences at a significance level of *α* = 0.05

AMF (*v*/*v* %)	Date (days)	N form	PRC (%)	Probability level
0			0 ± 0 c	*0.007* (0 vs. 0.3)
0.3			19.04 ± 8.3 b	*< 0.001* (0 vs. 3)
3.0			31.88 ± 9.2 a	*0.013* (0.3 vs. 3)
	0		21.23 ± 7.4	0.502
	65		12.71 ± 1.3	
		NO_3_^−a^	32.51 ± 7.5 a	*< 0.001*
		NH_4_^+b^	1.44 ± 5.5 b	
0		NO_3_^−^	0 ± 0^c^	*< 0.001* (0 vs. 0.3)
0.3			38.09 ± 12.6	*0.014* (0.3 vs. 3)
3.0			59.43 ± 7.4	
0		NH_4_^+^	0 ± 0	*0.004* (0 vs. 3)
0.3			0 ± 0	*< 0.001* (0.3 vs. 3)
3.0			4.33 ± 3.8	

^a^NO_3_^−^ predominant = 95% NO_3_^−^/5% NH_4_^+^

^b^NH_4_^+^ predominant = 25% NO_3_^−^/75% NH_4_^+^

^c^Only the results of the AMF × N form treatments are presented averaged over the dates

### Plant growth and mineral nutrient uptake

Inoculation with the low AMF amount of 0.3% resulted in lower bulb fresh and dry mass than those of non-inoculated plants (Table 2). The time of inoculation had no effect on bulb mass. Bulb mass at predominant NO_3_^−^ compared with NH_4_^+^ supply was enhanced. Onion plants had the highest bulb fresh and dry mass (49.9 ± 7.1 and 7.89 ± 1.2 g) with predominant NO_3_^−^. Dry matter of the bulbs was reduced at predominant NH_4_^+^ versus predominant NO_3_^−^ supply. Moreover, reduced dry matter content was observed in the AMF-inoculated bulbs in combination with predominant NH_4_^+^ supply.

**Table 2:** Effect of mycorrhizal inoculation (AMF), date of inoculation (days after seeding), and nitrogen (N) form on fresh mass (FM), dry mass (DM), and dry matter content (DMC) of onion plants (*cv*. Stuttgarter Riesen) analyzed after a growing period of 149 days in a three factorial ANOVA based on an orthogonal design with six replications. Means ± standard deviation followed by the same letter do not differ significantly according to Duncan’s multiple range test. Probability levels written in italics indicate significant differences at a significance level of *α* = 0.05

AMF (*v*/*v* %)	Date (days)	N form	FM (g plant^−1^)	DM (g plant^−1^)	DMC (gkg-1)
0			45.7 ± 8.5 a	6.84 ± 1.7 a	14.8 ± 1.2
0.3			40.6 ± 9.6 b	5.91 ± 1.8 b	14.4 ± 1.8
3.0			43.5 ± 10.2 ab	6.43 ± 2.0 ab	14.6 ± 1.5
	0		42.3 ± 9.9	6.22 ± 1.9	14.5 ± 1.5
	65		44.2 ± 9.4	6.57 ± 1.8	14.7 ± 1.6
		NO_3_^–a^	49.9 ± 7.1 a	7.89 ± 1.2 a	15.8 ± 0.64
		NH_4_^+b^	36.7 ± 6.9 b	4.90 ± 1.0 b	13.4 ± 1.2
0		NO_3_^−^	51.4 ± 6.1^c^	8.07 ± 1.3^c^	15.0 ± 0.79 a^c^
0.3			46.8 ± 8.3	7.46 ± 1.2	16.0 ± 0.61 a
3.0			51.6 ± 6.4	8.18 ± 1.2	15.8 ± 0.48 a
0		NH_4_^+^	40.4 ± 7.2	5.71 ± 1.2	14.0 ± 0.91 b
0.3			34.4 ± 6.7	4.36 ± 0.65	12.8 ± 1.2 c
3.0			35.2 ± 5.8	4.63 ± 0.55	13.3 ± 1.2 c
Source of variation			Probability level		
AMF			*0.044*	*0.014*	0.364
Date			0.253	0.175	0.252
N form			*< 0.001*	*< 0.001*	*< 0.001*
AMF × date			0.492	0.448	0.505
AMF × N form			0.336	0.108	*0.010*
Date × N form			0.502	0.550	0.617
AMF × date × N form			0.275	0.737	0.312

^a^NO_3_^−^ predominant = 95% NO_3_^−^/5% NH_4_^+^

^b^NH_4_^+^ predominant = 25% NO_3_^−^/75% NH_4_^+^

^c^Only the results of the AMF × N form interaction are presented averaged over the dates

Mycorrhizal treatments resulted in a significant increase in nitrogen and potassium content in bulbs while the content of phosphorus was not significantly influenced (Table 3). The higher the inoculum amount the higher the nitrogen and potassium content. Predominant NH_4_^+^ supply increased nitrogen and phosphorus content compared with predominant NO_3_^−^, whereas the highest potassium content was observed in bulbs treated with predominant NO_3_^−^ and high inoculum. The time of inoculation did not affect nutrient content at all because biomass increased when inoculated at the second date but nutrient concentrations decreased significantly. In all other cases, when nutrient content increased, it was because of a gain in the nitrogen or phosphorus concentrations.

**Table 3:** Effect of mycorrhizal inoculation (AMF), date of inoculation (days after seeding) and nitrogen (N) form on nitrogen, phosphor (P), and potassium (K) content (% in the dry mass of onion bulbs (*cv*. Stuttgarter Riesen) analyzed after a growing period of 149 days in a three factorial ANOVA based on an orthogonal design with six replications. Means ± standard deviation followed by the same letter do not differ significantly according to Duncan’s multiple range test. Probability levels written in italics indicate significant differences at a significance level of *α* = 0.05

AMF (*v*/*v* %)	Date (days)	N form	N (mg g^−1^)	N (g plant^−1^)	P (mg g^−1^)	P (g plant^−1^)	K (mg g^−1^)	K (g plant^−1^)
0			20.65 ± 1.2 b	1.24 ± 0.28 b	2.33 ± 0.30 b	0.15 ± 0.02	16.90 ± 1.6	0.99 ± 0.29 b
0.3			21.62 ± 1.3 a	1.33 ± 0.32 ab	2.66 ± 0.42 a	0.15 ± 0.03	17.00 ± 1.58	1.07 ± 0.34 ab
3.0			21.47 ± 1.9 a	1.39 ± 0.25 a	2.62 ± 0.41 a	0.15 ± 0.04	16.91 ± 0.99	1.15 ± 0.26 a
	0		21.90 ± 1.4 a	1.32 ± 0.31	2.65 ± 0.42 a	0.16 ± 0.04	17.44 ± 2.01 a	1.06 ± 0.3
	65		20.60 ± 1.7 b	1.32 ± 0.26	2.43 ± 0.33 b	0.15 ± 0.03	16.43 ± 0.98 b	1.08 ± 0.3
		NO_3_^–a^	19.72 ± 1.5 b	1.11 ± 0.19 b	2.37 ± 0.33 b	0.13 ± 0.02	16.99 ± 1.10	1.33 ± 0.18 a
		NH_4_^+b^	22.77 ± 1.7 a	1.54 ± 0.19 a	2.70 ± 0.40 a	0.18 ± 0.02	16.88 ± 1.78	0.82 ± 0.14 b
0	0	NO_3_^−^	20.30 ± 1.3 cde	1.50 ± 0.17^c^	2.33 ± 0.22 bc	0.17 ± 0.015 b^c)^	17.66 ± 1.36	1.30 ± 0.13^c^
0.3			20.20 ± 1.9 def	1.47 ± 0.19	2.45 ± 0.36 bc	0.18 ± 0.02 ab	17.0 ± 0.89	1.24 ± 0.19
3.0			20.50 ± 1.7 cde	1.61 ± 0.19	2.66 ± 0.29 ab	0.19 ± 0.029 a	16.66 ± 1.21	1.38 ± 0.19
0	0	NH_4_^+^	22.16 ± 1.1 bc	1.23 ± 0.23	2.48 ± 0.23 bc	0.12 ± 0.014 c	17.16 ± 1.72	0.93 ± 0.17
0.3			24.66 ± 1.9 a	1.11 ± 0.13	3.02 ± 0.39 a	0.13 ± 0.024 c	18.16 ± 1.47	0.75 ± 0.08
3.0			23.33 ± 1.3 ab	1.17 ± 0.13	2.95 ± 0.26 a	0.13 ± 0.019 c	17.66 ± 0.81	0.77 ± 0.11
0	65	NO_3_^−^	18.60 ± 1.2 f		2.12 ± 0.36 c		16.66 ± 1.21	
0.3			19.50 ± 1.5 ef		2.51 ± 0.31 bc		16.33 ± 1.03	
3.0			19.0 ± 0.89 ef		2.16 ± 0.12 c		17.16 ± 0.75	
0	65	NH_4_^+^	21.50 ± 1.5b bcd		2.41 ± 0.27 bc		16.0 ± 1.67	
0.3			22.16 ± 0.9 bc		2.66 ± 0.47 ab		16.5 ± 2.25	
3.0			22.83 ± 1.7 b		2.70 ± 0.48 ab		15.83 ± 1.83	
Source of variation			Probability level					
AMF			*0.050*	*0.025*	*0.004*	0.22	0.972	*0.003*
Date			*< 0.001*	0.963	*0.01*	0.305	*0.006*	0.682
N form			*< 0.001*	*< 0.001*	*< 0.001*	*< 0.001*	0.765	*< 0.001*
AMF × date			0.854	0.437	0.410	0.113	0.928	0.258
AMF × N form			0.368	0.105	0.652	*0.050*	0.275	0.154
Date × N form			0.824	0.215	0.981	0.180	0.123	0.862
AMF × date × N form			*0.034*	0.577	*0.049*	0.624	0.622	0.094

^a^NO_3_^−^ predominant = 95% NO_3_^−^/5% NH_4_^+^

^b^NH_4_^+^ predominant = 25% NO_3_^−^/75% NH_4_^+^

^c^Only the results of the AMF × N form interaction are presented averaged over the dates

### Flavonol profile

The general flavonol profile had the highest abundance of QDG (71.9 μmol g^−1^; 67% of all flavonols), followed by QMG (33.6 μmol g^−1^; 30%) and IMG (4.19 μmol g^−1^; 4%) (Table 4). None of the treatments changed the composition of the three flavonols significantly. All treatments, however, interactively affected QDG and QMG in onion bulbs. A significantly higher QDG concentration was found in bulbs inoculated with the high AMF amount at the second inoculation date in combination with predominant NH_4_^+^ than for plants with the low AMF amount at the same date or with predominant NO_3_^−^ at the first date or not inoculated. QMG in bulbs also from the second inoculation date treated with the high amount of AMF inoculum at predominant NH_4_^+^ was significantly higher than the concentrations in bulbs from non- or low-inoculated plants at predominant NH_4_^+^ or at predominant NO_3_^−^ but only if inoculated before seeding. IMG was not affected by the inoculation date but was affected by the interaction of the amount of inoculum and nitrogen form. IMG increased significantly in inoculated versus non-inoculated plants at predominant NH_4_^+^ supply, and also in the treatments with the low inoculum amount at predominant NO_3_^−^ versus high or non-inoculated plants.

**Table 4:** Effect of mycorrhizal inoculation (AMF), date of inoculation (days after seeding), and nitrogen (N) form on the most important flavonols in bulbs of onion plants (*cv*. Stuttgarter Riesen) in a three-factorial ANOVA based on an orthogonal design with six replications. Means ± standard deviation followed by the same letter do not differ significantly according to Duncan’s multiple range test. Probability levels written in italics indicate significant differences at a significance level of *α* = 0.05

AMF (*v*/*v* %)	Date (days)	N form	QDG^c^ (μmol g^−1^)	QMG ^d^ (μmol g^−1^)	IMG^e^ (μmol g^−1^)
0			69.1 ± 17.1	30.9 ± 11.2	4.20 ± 1.3
0.3			67.4 ± 22.7	29.4 ± 11.0	3.90 ± 1.8
3.0			79.1 ± 32.0	40.9 ± 16.8	4.40 ± 2.2
	0		65.9 ± 19.1	30.2 ± 11.4	4.23 ± 1.8
	65		77.9 ± 27.7	37.0 ± 14.9	4.15 ± 1.8
		NO_3_^–a^	69.5 ± 24.8	33.4 ± 14.0	3.67 ± 1.6
		NH_4_^+ b^	74.3 ± 24.2	33.7 ± 13.7	4.51 ± 1.9
0	0	NO_3_^−^	62.4 ± 18.7 bc	30.7 ± 8.8 bc	3.72 ± 1.4 bc^f^
0.3			51.9 ± 8.27 c	21.0 ± 5.19 c	5.05 ± 2.2 a
3.0			71.4 ± 34.4 abc	38.3 ± 12.3 abc	3.43 ± 1.6 d
0	0	NH_4_^+^	66.6 ± 15.0 bc	26.6 ± 10.8 bc	3.42 ± 1.1 d
0.3			69.0 ± 22.9 abc	31.2 ± 19.7 bc	4.38 ± 1.4 b
3.0			74.1 ± 25.3 abc	33.0 ± 12.1 bc	4.64 ± 1.9 ab
0	65	NO_3_^−^	74.1 ± 16.2 abc	33.8 ± 17.8 bc	
0.3			85.5 ± 21.6 ab	37.5 ± 12.4 abc	
3.0			71.7 ± 24.6 abc	39.3 ± 13.1 ab	
0	65	NH_4_^+^	73.4 ± 7.19 abc	32.5 ± 5.42 bc	
0.3			63.2 ± 26.9 bc	27.8 ± 7.44 bc	
3.0			99.4 ± 37.6 a	50.9 ± 18.4 a	
Source of variation			Probability level		
AMF			*0.031*	*0.009*	0.725
Date			*0.033*	*0.025*	0.859
N form			0.382	0.940	0.153
AMF × date			0.934	0.804	0.856
AMF × N form			0.400	0.742	*0.042*
Date × N form			0.561	0.984	0.786
AMF × date × N form			*0.044*	*0.048*	0.129

^a^NO_3_^−^ predominant = 95% NO_3_^−^/5% NH_4_^+^

^b^NH_4_^+^ predominant = 25% NO_3_^−^/75% NH_4_^+^

^c^QDG = quercetin-3,4′-di-*O*-*β*-d-glucoside

^d^QMG = quercetin-4′- *O*-*β*-d-glucoside

^e^IMG = isorhamnetin-4′-*O*-*β*-d-glucoside

^f^Only the significant results of the AMF × N form interaction are presented averaged over the dates

### *CHS*, *FLS*, and *PAL* gene expression

In general, total abundance of all transcripts in inoculated plants was higher than in not inoculated plants, and it was higher at the second inoculation compared to the first inoculation date (Table 5). But no main factor influenced gene expression significantly (Table 5). *CHS1* and *FLS1* expressions were influenced by interactions between nitrogen form and amount of mycorrhizal inoculation, however, while *PAL1* expression was affected by the interaction of all three factors. Transcript levels of the *CHS1* gene were higher when inoculated than when not inoculated, but significantly so only with the low inoculum amount at predominant NO_3_^−^ and the high inoculum amount at predominant NH_4_^+^ supply independent of the date of inoculation. *FLS1* expression was higher in all treatments compared with non-inoculated at predominant NH_4_^+^, also independent of the date of inoculation. The highest expression was also found with the low inoculum amount at predominant NO_3_^−^ but it differed significantly only from the non-inoculated treatment at predominant NH_4_^+^. *PAL1* gene expression was highest with the low inoculum amount at predominant NO_3_^−^ after the first and second inoculation and at predominant NH_4_^+^ after the second inoculation and after the first inoculation with the high inoculum amount. In general, total abundance of all transcripts at the second inoculation date was higher than at the first inoculation.

**Table 5:** Effect of mycorrhizal inoculation (AMF), date of inoculation (days after seeding), and nitrogen (N) form on chalcone synthase (*CHS1*), flavonol synthase (*FLS1*), and phenylalanine ammonia lyase (*PAL1*) gene expression (relative based on CT values) of onion bulbs (*cv*. Stuttgarter Riesen) in a three-factorial ANOVA based on an orthogonal design with three replications. Values represent gene transcript abundance of *CHS1*, *FLS1*, and *PAL1* genes normalized to the reference gene *ALL ITS1* derived of three independent repetitions. Means ± standard deviation followed by the same letter do not differ significantly according to Duncan’s multiple range test. Probability levels written in italics indicate significant differences at a significance level of *α* = 0.05

AMF (*v*/*v* %)	Date (days)	N form	*CHS1*	*FLS1*	*PAL1*
0			2.83 ± 0.94	3.83 ± 1.79	2.43 ± 0.98
0.3			3.61 ± 1.06	4.54 ± 2.07	2.89 ± 1.76
3.0			2.95 ± 1.37	4.05 ± 1.23	2.44 ± 1.33
	0		2.76 ± 1.41	3.96 ± 2.01	2.21 ± 1.16
	65		3.51 ± 1.6	4.32 ± 1.69	2.97 ± 1.26
		NO_3_^–a^	3.32 ± 1.26	4.51 ± 1.67	2.65 ± 1.22
		NH_4_^+ b^	2.95 ± 1.32	3.78 ± 1.69	2.53 ± 1.23
0	0	NO_3_^−^	2.73 ± 1.2 b^c^	4.86 ± 1.09ab^c^	2.09 ± 0.93 b
0.3			4.56 ± 0.71 a	5.24 ± 1.5 a	4.16 ± 0.31 a
3.0			2.65 ± 0.7 b	3.40 ± 0.69 ab	2.09 ± 0.68 b
0	0	NH_4_^+^	2.92 ± 0.44 b	2.81 ± 0.43 b	2.38 ± 0.71 b
0.3			2.67 ± 1.68 b	3.85 ± 1.36 ab	−0.62 ± 3.37 c
3.0			3.26 ± 1.84 ab	4.70 ± 1.36 ab	3.15 ± 2.09 ab
0	65	NO_3_^−^			2.30 ± 0.71 b
0.3					3.39 ± 0.9 ab
3.0					1.84 ± 0.77 b
0	65	NH_4_^+^			2.94 ± 0.23 ab
0.3					4.64 ± 0.64 a
3.0					2.69 ± 1.68 b
Source of variation			Probability level		
AMF			0.237	0.528	0.710
Date			0.065	0.498	0.147
N form			0.347	0.177	0.821
AMF × Date			0.454	0.165	0.126
AMF × N form			*0.035*	*0.030*	0.092
Date × N form			0.517	0.690	0.054
AMF × Date × N form			0.063	0.202	*0.039*

^a^NO_3_^−^ predominant = 95% NO_3_^−^/5% NH_4_^+^

^b^NH_4_^+^ predominant = 25% NO_3_^−^/75% NH_4_^+^

^c^Only the significant results of the AMF × N form interaction are presented averaged over the dates

## Discussion

### Mycorrhization, plant growth, and nutrient uptake

Plant genetic characteristics as well as environmental factors related to the soil, such as properties, texture, soil pH, and nutrient concentration and distribution, directly affect nutrient availability and indirectly soil microorganisms including AMF (Carrenho et al. 2007). AMF colonization is influenced by additional factors, such as plant species, inoculum density, inoculum type, root growth rates, root age, and colonization period (McGonigle 2001). Although Daft and Nicolson (1969) used different amounts of inocula of *Endogone macrocarpa* for tomato, they found a similar percentage root length colonized 84 days after inoculation. In contrast, our plants inoculated with high amounts of inoculum showed a higher percentage root length colonized at harvest than plants treated with low amounts of inoculum. This was the case also for the second inoculation that was followed by 84 days of subsequent growing period. It can be assumed that the inoculum potential and days until harvest were not sufficient for the low inoculum amount (0.3%) to have attained asymptotic colonization (McGonigle 2001). In agreement with our results, an enhanced growth of tomato plants, e.g., larger leaves, was associated with an elevated inoculum amount (Daft and Nicolson 1969). When our onion plants were inoculated 65 days after seeding, mycorrhization occurred, but a high amount of inoculum did not colonize significantly more roots than the low amount. Besides the reasons mentioned previously, this could also be because of other factors, such as (i) a high portion of old roots with few root tips as a primary entrance for the fungi (Smith and Walker 1981), (ii) effective “dilution” of inoculum at the second inoculation date across an already established a root system (Tawaraya et al. 1999), (iii) plant needs of assimilates obtained from photosynthesis to attain a high potential growth rate and to store for bulb formation, and (iv) competition among plants grown together in one pot (Hodge 2009). Moreover, AMF colonization decreased with predominant NH_4_^+^ supply. Nitrogen form can affect AMF colonization by modifying the soil pH (Perner et al. 2008). Nitrate assimilation is known to be a proton-consuming process while NH_4_^+^ assimilation is considered a proton-producing process. Predominant NH_4_^+^ causes acidification of the rhizosphere resulting in slower root penetration and low AMF colonization in consequence (Graw 1979; Mengel and Kirkby 2001; Shen et al. 2005). Plant growth decreased with predominant NH_4_^+^ supply as well, which is in agreement with previous findings (Perner et al. 2008). Another reason for the decreased bulb growth and low dry matter content at predominant NH_4_^+^ supply could be a reduced activity of NO_3_^−^ reductase in the leaves in response to NH_4_^+^ accumulation (Jalloh et al. 2009). The depressive effect of NH_4_^+^ on NO_3_^−^ reductase activity is associated with NH_4_^+^ accumulation and pH decrease (Marschner 2012).

All pots in our experiment were provided with sufficient nutrients and no deficiency was observed. Nevertheless, in our experiment AMF stimulated total nutrient uptake (nitrogen and potassium) and also increased nutrient concentrations (nitrogen and phosphorus) in bulb dry mass, which also is known from experiments of other investigators (George 2000; Janos et al. 2001; Perner et al. 2008). This might be because of the increment of AMF and their ability to facilitate uptake of several nutrients, such as P, N, K, Ca, Mg, Na, and Zn, by changing root architecture and forming a network of fungal hyphae in the soil (Chu 1999; Liu et al. 2002; Lenin et al. 2010). Increased nitrogen and phosphorus concentrations with predominant NH_4_^+^ fertilization in our study are based on an increased active anion uptake (Jing et al. 2010). In turn, decreased potassium uptake at predominant NH_4_^+^ supply suggest an antagonistic effect between NH_4_^+^ and potassium, which could result from competition between potassium and NH_4_^+^ for transporters (Mengel and Kirkby 2001; Milford and Johnston 2007). Although, phosphorus concentration increased at predominant NH_4_^+^ supply, higher phosphorus content in plants inoculated with a high amount of inoculum together with predominant NO_3_^−^ fertilization can be attributed to high root colonization leading to both enhanced growth and NO_3_^−^ uptake.

### Flavonols, *PAL*, *CHS*, and *FLS* gene expression

In the present study, the composition and concentrations of the major onion flavonols, namely QDG, QMG, and IMG, are in accordance with the findings of other authors (Price and Rhodes 1997; Marotti and Piccaglia 2002; Mollavali et al. 2016). Similar to those studies, QDG concentration in our results was about twofold higher than QMG concentration, and IMG at about 4% amounted to the smallest portion of the total flavonol content. Interestingly and in contrast to the data of others (Perner et al. 2008; Fallovo et al. 2011; Osuagwu and Edeoga 2012; Zaghdoud et al. 2016), the two nitrogen forms solely did not affect flavonol concentration in our experiment but did so in interaction with AMF inoculation, confirming the importance of the environmental conditions for plant nutrition. Results in the literature are contradictory. On one hand, if the competition between polyphenolic compounds and protein biosynthesis is decreased, which can be caused by a predominant NH_4_^+^ supply also possibly resulting in cell toxicity, a higher concentration of flavonols is expected (Ibrahim et al. 2012; Zaghdoud et al. 2016). On the other hand, an advantage of a NO_3_^−^ over an NH_4_^+^ supply led to increasing quercetin and isorhamnetin concentrations (Perner et al. 2008; Fallovo et al. 2011). In the experiments of Fallovo et al. (2011), flavonol concentration increases happened only when *Brassica* plants had insufficient light (< 5 mol m^−2^ d^−1^). Under increased radiation, nitrogen forms did not affect flavonoids. Consequently, other limiting conditions in addition to low light, such as low nutrient supply (Koeslin-Findeklee et al. 2015), or low temperature (Watanabe and Ayugase 2015) may interact with the effect of nitrogen nutrition. In our study, onions likely had no nitrogen deficiency whatsoever because the tissue concentration of total nitrogen was comparable with data from Huett et al. (1997). Moreover, plants received a 75% NH_4_^+^ supply and were cultivated under high daily light levels of 29 mol m^−2^ and a suitable temperature of 21/17 °C (day/night). This might explain the missing effect of the nitrogen form. We found no significant correlation between nitrogen tissue concentration and single or total flavonols (data not shown).

Flavonol concentration in our experiment did increase significantly as a result of a late AMF inoculation (65 days) with the high amount of inoculum (3%) at predominant NH_4_^+^ supply. This supports the hypothesis that AMF colonization during the early phase of the symbiosis may induce higher flavonol concentration as a defense response, as was described previously (Guo et al. 2006, 2007; Yao et al. 2007; Perner et al. 2008). Such defense reactions are mostly known from attacks of fungal pathogens (reviewed by Rao 1990; Steinkellner et al. 2007; Hassan and Mathesius 2011). Indeed, some flavonols act as regulatory signals for the susceptibility of roots to AMF at the beginning of formation of the symbiosis as has been shown for quercetin stimulating the hyphal growth of different AMF genera (Bécard et al. 1992). After the early stage of the symbiosis, however, fungi may favor a change of the concentrations of specific flavonols, although the flavonol composition in our data was not affected by colonization. These data reflect only one time point (7 days after the second inoculation) and, therefore, are not sufficient to support this inference. Nevertheless, flavonol concentration at harvest increased more after the second inoculation treatment than after inoculation at seeding. Mycorrhizal symbiosis can stimulate flavonol accumulation both to establish the symbiosis and as a consequence of biotic stress (Larose et al. 2002). The low amount of AMF inoculum used in our treatments does not seem to be sufficient to trigger a defense response. Besides, after the symbiosis is established for an extended time after inoculation, the production of flavonoids as phytoanticipines is no longer necessary (Slimestad et al. 2007). In contrast to quercetin, IMG concentration was not affected by AMF inoculation, as also was shown by Perner et al. (2008). Although the increase in flavonols is most intense with predominant NH_4_^+^ fertilization, the effect seems to be somewhat temporary. The results regarding the interaction between the nitrogen form, AMF inoculation, and date of inoculation do not support our hypothesis that a predominant NH_4_^+^ supply induces a persistent effect on flavonols.

*PAL* previously was found to be highly regulated at the transcriptional level in response to nitrogen source and abiotic factors (Olsen et al. 2008). In the present study, *PAL1* expression was higher with predominant NH_4_^+^ than NO_3_^−^ supply, which is in agreement with previous studies demonstrating that increased activity of *PAL* in NH_4_^+^-induced tissue can be due to the secondary nitrogen assimilation pathway activation through phenylpropanoid metabolism (Singh et al. 1998, Mihaljević et al., 2011). Some studies have shown that high C/N ratios resulted in an increase in the concentration of flavonols and also transcript levels of the flavonoid pathway genes (Martin et al. 2002, Wan et al. 2015). Increased *PAL* expression in our study could be a result of both higher C/N ratio in AMF inoculated plants related to enhanced photosynthesis rate and reduced C allocation from the plant to the fungi due to the nitrogen supply (Miller et al. 2002, Blanke et al. 2005). Some authors observed high levels of *PAL* and *CHS* transcripts in roots colonized by AMF (Harrison and Dickson 1994; Manibhushan and Manian 1995; Bonanomi et al. 2001). Results of our study indicate that the expression of *CHS* and *FLS*, two key enzymes in flavonoid biosynthesis (Petrussa et al. 2013), was induced in onion plants inoculated with a low amount of inoculum and supplied with predominant NO_3_^−^ (Moche et al. 2010). This suggests that under certain conditions, mycorrhizal colonization has the potential to contribute to a persistent increase in flavonols. Moreover, the activity of enzymes involved in the flavonoid pathway may depend on the nitrogen source (Fujiwara et al. 2015). Bonanomi et al. (2001) discovered an induction of a *CHS* gene (*Mt-CHS1*) after inoculation of *Medicago truncatula* with *Glomus intraradices*. They measured the expression on several different days post inoculation (5, 10, 15, and 20 dpi). While the induction always was found at 5 dpi, it could not be detected continuously at the later dates, which is in agreement with other reported results (Volpin et al., 1995; Mohr et al. 1998). The authors concluded that the expression was particularly detectable at the stage of the first contact with *G. intraradices*. Our sampling date for the measurement of gene expressions was 7 days after the second inoculation, a similar period to those chosen by Bonanomi et al. (2001). Plants from the first inoculation had their first contact with AMF 65 days earlier, however, and our plant material was different as we used bulbs and the others roots which might explain why we did not find a significant expression pattern.

Data from the present study provide evidence that a predominant NH_4_^+^ supply together with AMF inoculation can significantly increase the content of flavonols in onion bulbs. AMF inoculation may act as biotic stress in the early stage of the symbiosis. To confirm this indication, measurements of expressions of related genes have to be carried out through time (Bonanomi et al. 2001). Although our data indicate there might be a permanent increase in flavonols under certain conditions, an increase of defense-related flavonoids can hardly be expected once the symbiosis is established. Nevertheless, an increase of flavonols in connection with AMF inoculation persisted at least 84 days after our second inoculation treatment.
